# A Comprehensive Review on Exploring the Impact of Telemedicine on Healthcare Accessibility

**DOI:** 10.7759/cureus.55996

**Published:** 2024-03-12

**Authors:** Pankajkumar A Anawade, Deepak Sharma, Shailesh Gahane

**Affiliations:** 1 Management, School of Allied Sciences, Datta Meghe Institute of Higher Education & Research, Wardha, IND; 2 Science and Technology, School of Allied Sciences, Datta Meghe Institute of Higher Education and Research, Wardha, IND

**Keywords:** health outcomes, technology adoption, health equity, geographical barriers, healthcare accessibility, telemedicine

## Abstract

Telemedicine has emerged as a transformative force in healthcare delivery, particularly in improving healthcare accessibility. This comprehensive review examines the impact of telemedicine on healthcare accessibility, exploring its ability to overcome geographical, financial, sociocultural, and infrastructural barriers to healthcare access. Through remote consultations, monitoring, and diagnosis facilitated by technology, telemedicine extends healthcare reach to remote and underserved areas while enhancing temporal accessibility with round-the-clock availability. By streamlining healthcare delivery systems, telemedicine reduces costs and promotes efficiency, ultimately fostering health equity and improving health outcomes. However, technological barriers, regulatory hurdles, and patient acceptance remain. To realize telemedicine's full potential, collaboration among stakeholders in the healthcare and technology sectors is imperative. Policymakers must enact supportive regulations, healthcare providers must integrate telemedicine into their practices, and technology companies must innovate to develop user-friendly platforms. Through concerted efforts, telemedicine can catalyze advancing healthcare accessibility and enhance the health and well-being of individuals worldwide.

## Introduction and background

Telemedicine refers to the provision of healthcare services remotely through the use of telecommunications and information technology. This can include video consultations, remote monitoring, and electronic transmission of medical records. The significance of telemedicine lies in its ability to bridge geographical barriers, improve patient access to healthcare, and enhance the efficiency of healthcare delivery systems. By enabling healthcare professionals to diagnose, treat, and monitor patients from a distance, telemedicine can revolutionize how healthcare is delivered, particularly in areas with limited access to medical facilities [1].

Healthcare accessibility refers to the ease with which individuals can obtain timely and appropriate healthcare services. Access to healthcare is a fundamental human right for promoting health equity and addressing health disparities. However, numerous geographical, financial, cultural, and infrastructural barriers can limit individuals' ability to access healthcare services. Ensuring healthcare accessibility is crucial for improving health outcomes, reducing healthcare costs, and promoting social and economic development [2].

The purpose of this review is to explore the impact of telemedicine on healthcare accessibility. By examining existing literature, case studies, and empirical evidence, this review aims to elucidate how telemedicine can address barriers to healthcare access and improve the overall accessibility of healthcare services. In addition, this review will identify challenges and limitations associated with the adoption of telemedicine and provide recommendations for policymakers, healthcare providers, and other stakeholders to maximize the potential benefits of telemedicine in promoting healthcare accessibility.

## Review

Evolution of telemedicine

Historical Overview

Telemedicine has a long history, dating back over a century. The practice of telemedicine closely follows the evolution of communication and information technologies. In the early 20th century, the inventions of communication tools, such as the telegraph and telephone, jumpstarted the practice of telemedicine. In 1905, heart sounds were transmitted using the telephone, and in 1910, electrocardiography and remote diagnosis were achieved. The use of telegraphs and telephones in the military during the Civil War also contributed to the early development of telemedicine. The modern concept of telemedicine, as we know it today, emerged in the late 1960s, with projects led by NASA and the Nebraska Psychology Institute, which resulted in the medium of closed-circuit television being used for remote monitoring and healthcare consultations [3-5]. The widespread use of telephones, the Internet, and modern digital technologies further propelled the evolution of telemedicine. Today, telemedicine has become an integral part of healthcare, especially with the increased reliance on telemedicine and telehealth services during the COVID-19 pandemic. Modern equipment, such as wearable devices and digital cameras, has further enhanced the delivery of remote healthcare services [6,7].

Technological Advancements

Telemedicine has made significant strides across various domains of healthcare delivery, leveraging technological innovations to enhance patient care and accessibility. In the realm of surgical procedures, advancements in telemedicine have revolutionized traditional practices. Surgeons can now conduct remote surgical consultations, enabling them to provide expert guidance and support regardless of geographical barriers. Telementoring has emerged as a valuable tool for skill development, allowing experienced surgeons to mentor their peers in real time, fostering knowledge transfer and expertise sharing. Moreover, the advent of telerobotic surgery has pushed the boundaries of surgical capabilities, enabling surgeons to perform intricate procedures from remote locations with the assistance of robotic systems, thereby expanding access to specialized surgical care [8]. Diagnostic telemedicine has similarly transformed the diagnostic process, particularly by remotely interpreting medical images, such as X-rays and MRIs. Telemedicine platforms facilitate the seamless transmission of medical images, enabling radiologists and specialists to analyze images remotely and provide timely diagnostic assessments. In addition, telepathology has emerged as a vital component of diagnostic telemedicine, allowing pathologists to examine tissue samples remotely and make diagnostic interpretations. This remote analysis enhances diagnostic accuracy and expedites treatment decisions, particularly in underserved areas where access to pathology expertise may be limited [8].

Advanced telemedicine devices and communication systems have greatly enhanced the remote monitoring of patients. These technologies enable healthcare providers to remotely monitor patients' vital signs, medication adherence, and disease progression, facilitating proactive interventions and personalized care plans. Furthermore, telemedicine fosters ongoing communication between patients and healthcare providers, promoting patient engagement and empowerment. Through remote consultations and virtual visits, individuals can access timely medical advice and support from healthcare professionals, regardless of location. This continuous monitoring and support enhance patient outcomes and satisfaction while reducing the burden on traditional healthcare facilities [8]. The advent of the Internet has played a pivotal role in advancing telemedicine capabilities, facilitating the transmission of medical data over long distances. Healthcare providers can securely exchange patient information, medical records, and diagnostic images through telecommunication channels, enabling collaborative decision-making and coordinated care delivery. Moreover, the development of healthcare applications and telemedicine platforms has streamlined the exchange and storage of medical information, enhancing accessibility and efficiency in healthcare delivery. These Internet-based technologies have democratized access to healthcare services, breaking down geographical barriers and expanding the reach of medical expertise to remote and underserved populations [6].

The evolution of telemedicine has necessitated the development of robust regulatory and security measures to safeguard patient privacy and data security. Compliance with HIPAA regulations ensures patient information confidentiality and protects against unauthorized access or disclosure. In addition, secure information transmission protocols and encryption technologies are employed to prevent data breaches and ensure the integrity of transmitted data. While these regulatory and security measures are essential for maintaining patient trust and confidentiality, they also present challenges in terms of interoperability, standardization of communication methods, and policy changes. Addressing these challenges requires collaborative efforts from policymakers, healthcare providers, and technology developers to establish uniform standards and guidelines that facilitate the widespread adoption and utilization of telemedicine technologies [6,9,10].

Current State of Telemedicine Adoption

The current status of telemedicine adoption in the United States demonstrates substantial and consistent growth. As trend analysis indicates, telehealth now constitutes approximately 10% of all outpatient clinic visits nationwide, marking a significant escalation from pre-pandemic levels and solidifying its position as a fundamental component within numerous clinical service lines and care models [11]. However, notable regional disparity exists in telehealth utilization, with specific areas exhibiting higher adoption rates, potentially influenced by state-level regulations [11]. Telehealth utilization rates were found to be lowest among individuals lacking insurance coverage, young adults aged 18 to 24, and residents of the Midwest [12]. The onset of the pandemic spurred a surge in telehealth adoption, particularly within underserved communities, highlighting the imperative of addressing access barriers for vulnerable populations, including those with disabilities, and necessitating substantial, enduring changes in technology, regulatory frameworks, and legislative infrastructure [13]. Moreover, the State of Telemedicine Report 2023 findings reveal that telemedicine adoption remained robust across all physician age demographics, with over 78% of physicians advocating for equitable compensation between telemedicine and in-person visits [14]. This persistent growth trajectory in telemedicine adoption is anticipated to persist, with telemedicine poised to assume a significant role in shaping the healthcare landscape in the foreseeable future [7].

Understanding healthcare accessibility

Definition and Dimensions of Healthcare Accessibility

Healthcare accessibility encompasses the ability of individuals to obtain necessary healthcare services when required, constituting a multifaceted concept incorporating various dimensions. These dimensions include affordability, accessibility, availability, accommodation, and acceptability [15-17]. Affordability pertains to the financial feasibility of healthcare services and individuals' capacity to cover associated costs. Accessibility concerns the geographical proximity of healthcare facilities and the ease of reaching them. Availability denotes the presence of healthcare resources, including personnel and technology, to adequately address patients' needs. Accommodation reflects how healthcare services are structured to accommodate patients' constraints and preferences. Acceptability evaluates healthcare services' cultural appropriateness and alignment with patients' expectations [15,17,18]. Disparities in healthcare access persist based on various factors such as race, ethnicity, socioeconomic status, age, gender, disability status, sexual orientation, gender identity, and residential location [19]. Addressing these disparities is imperative to ensure equitable access to quality healthcare for all individuals, including marginalized populations. This necessitates enhancements to healthcare infrastructure, policy frameworks, and resource allocation to bridge existing gaps [16,19].

Factors Influencing Healthcare Accessibility

The factors influencing healthcare accessibility are multifaceted, encompassing various dimensions, such as availability, affordability, acceptability, and appropriateness of care. Access to healthcare is defined as the opportunity to identify health needs and reach, obtain, and utilize healthcare services, ultimately meeting those needs. It can be conceptualized as a continuum, impacted by provider availability, appointment scheduling ease, financial capability to pay for care, and access to healthcare facilities [20]. Barriers to access encompass a wide array of challenges, ranging from limited health insurance coverage and transportation options to extended wait times for appointments and restricted availability of after-hours care [21]. Disparities in healthcare access persist based on numerous factors, such as race, ethnicity, socioeconomic status, age, gender, disability status, and residential location [19]. Particularly for individuals with disabilities, factors like availability, acceptability, affordability, and geographic proximity significantly influence their access to primary healthcare services, particularly in rural settings [2]. Addressing these multifaceted factors is essential to ensure equitable access to healthcare for all individuals, irrespective of their demographic background or geographical location.

Challenges in Achieving Healthcare Accessibility

Various factors impede access to healthcare, each contributing to the complex landscape of healthcare accessibility. High healthcare costs pose a formidable barrier, even for insured individuals, as exorbitant out-of-pocket expenses compel many to prioritize essential needs over medical care, ultimately avoiding necessary treatment [20]. In addition, transportation barriers exacerbate accessibility challenges, particularly in rural locales, where limited access to transportation infrastructure hinders individuals from reaching healthcare facilities, thereby obstructing their ability to obtain vital medical services [22]. Implicit biases and systemic discrimination within the medical community perpetuate health disparities by instilling fear and apprehension in marginalized populations, leading to healthcare avoidance and delayed treatment-seeking behavior [22]. Furthermore, while telemedicine presents opportunities for expanding healthcare access, it also introduces a new set of challenges, particularly for individuals with disabilities, including infrastructure limitations, operational hurdles, regulatory complexities, communication barriers, and legislative impediments [23].

**Figure 1:**
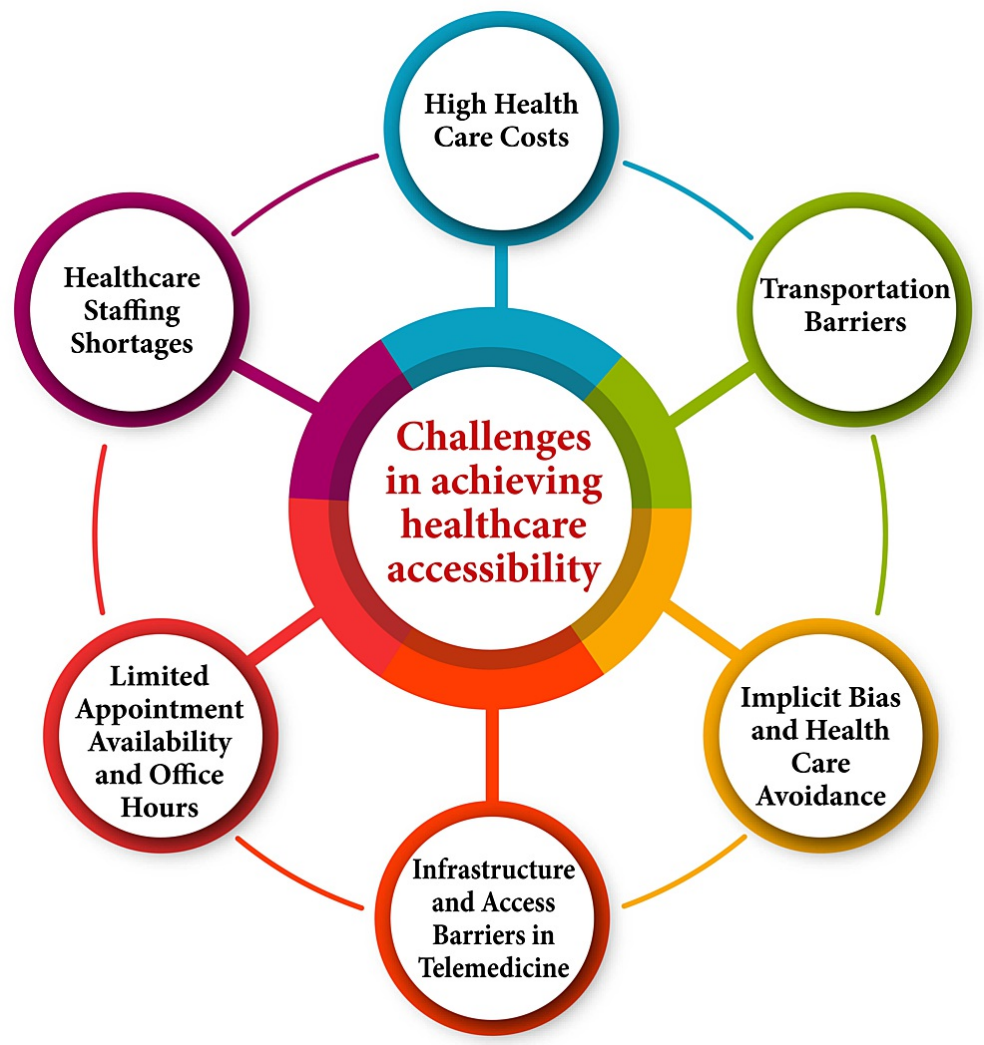
Challenges in achieving healthcare accessibility

Limited appointment availability and constrained office hours within healthcare organizations compound accessibility issues, rendering it arduous for working adults or caregivers to schedule timely appointments, consequently impeding their ability to access necessary care [24]. Compounding these challenges are healthcare staffing shortages, particularly in underserved areas, where deficits in primary care physicians and other healthcare professionals restrict individuals' access to essential medical services [25]. Moreover, inadequate insurance coverage remains a significant barrier to healthcare access, as insufficient coverage deprives many individuals of the financial means to seek necessary medical care, thus exacerbating health disparities and perpetuating inequities in healthcare access [25]. Collectively, these multifaceted barriers underscore the imperative of addressing systemic challenges and implementing comprehensive solutions to ensure equitable access to healthcare for all individuals. Challenges in achieving healthcare accessibility are shown in Figure 1.

Impact of telemedicine on healthcare accessibility

Improving Geographical Accessibility

Case studies/examples: Telemedicine has emerged as a pivotal tool in enhancing geographical accessibility to healthcare, offering significant potential to mitigate health disparities by granting individuals in rural or underserved areas access to essential medical services [26]. Reported benefits of telemedicine include reduced healthcare costs, enhanced medication reconciliation, and minimized exposure to communicable diseases, which is particularly crucial during public health crises, such as pandemics [23]. However, various challenges and barriers, encompassing infrastructure limitations, operational complexities, regulatory hurdles, communication obstacles, and legislative constraints, present significant obstacles, particularly for vulnerable populations, such as individuals with disabilities [23]. To bolster access to telehealth among underserved patients, healthcare providers must integrate strategies into their practice to engage and serve these communities, ensuring equitable access to essential telehealth services [27]. In addition, addressing issues like digital literacy, provision of necessary equipment, communication tools, and regulatory and legislative barriers is imperative to ensure fair and inclusive access to telemedicine for all individuals [23].

Statistics supporting the impact: In 2021, 37.0% of adults in the United States reported utilizing telemedicine within the preceding 12 months, with higher usage observed among women (42.0%) than men. Telemedicine utilization also demonstrated an age-related increase, and while usage was relatively consistent among individuals with family incomes below 100% of the federal poverty level (33.1%), it rose steadily with increasing family income, reaching 40.7% among those with family incomes exceeding 400% of the federal poverty level [28]. Advancing the frontiers of geographic accessibility to healthcare services is imperative to identify and address communities left underserved. Integrating geospatial technologies, such as global navigation satellite systems, geographical information systems, and earth observation, has facilitated numerous studies examining potential access disparities. Telemedicine has emerged as a prominent modality capable of enhancing access to healthcare services, particularly in remote or underserved regions [29]. To ensure health equity in telehealth, proactive measures are necessary to meet the needs of underserved populations through enhancements to telehealth workflows, staff training, and community resources. Achieving equal access to quality telehealth care for all individuals, including those from underserved backgrounds, necessitates a collective effort and shared responsibility [30].

Enhancing Temporal Accessibility

Real-time consultations: The integration of real-time consultations into telemedicine practices can significantly enhance temporal accessibility, offering patients the flexibility to access healthcare services at their convenience while minimizing the need for travel and associated time and cost burdens [31]. Leveraging real-time traffic information, healthcare providers can strategically allocate consulting hours across different periods, resulting in dynamic accessibility outcomes tailored to patient needs. Despite the potential benefits, numerous barriers and challenges hinder the widespread adoption of telemedicine practices, including infrastructure limitations, operational complexities, regulatory constraints, communication barriers, and legislative hurdles [32]. To address these challenges and ensure equitable access to telehealth care, healthcare providers must implement strategies to reach underserved populations, particularly vulnerable groups with limited digital literacy [27].

24/7 availability: Expanding temporal accessibility in healthcare through telemedicine entails the provision of 24/7 availability of healthcare services, effectively reducing patients' reliance on physical hospital or clinic visits for medical care [31]. Real-time traffic information can be utilized to calculate travel time, while a demand weight index can dynamically adjust population demand, optimizing healthcare accessibility across various periods [31]. Despite the potential benefits, multiple barriers and challenges impede the adoption of telemedicine practices, including infrastructure constraints, operational hurdles, regulatory complexities, communication obstacles, and legislative barriers [33]. Individuals with disabilities face additional barriers, including equipment and communication challenges, along with limited access to patient education materials tailored to their needs [23]. To mitigate these barriers, telemedicine platforms should incorporate customized features to facilitate healthcare communications for individuals with disabilities, while adherence to web accessibility standards is crucial to ensure inclusivity [23]. Furthermore, organizations can provide or loan devices to enable access to telehealth services, and digital literacy screening tools can aid in identifying the needs of individuals with disabilities [23,33].

Overcoming Financial Barriers

Cost-effectiveness of telemedicine: Telemedicine presents a promising avenue for delivering cost-effective outpatient care, particularly in rural regions where access to traditional healthcare services may be limited [34]. However, despite its potential benefits, barriers such as the upfront cost of telemedicine implementation and the inadequacies of existing payment models pose challenges to its widespread adoption, particularly in smaller rural hospitals where its impact could be most pronounced [35]. The cost-effectiveness of telemedicine hinges on three key factors: the distribution of costs between stakeholders, the efficacy of telemedicine in terms of patient satisfaction and successful clinical outcomes, and the indirect savings realized through reductions in patients' lost productivity [34]. Rural residents exhibit lower utilization rates of telemedicine services compared to their urban counterparts, with older individuals, those with lower incomes, and lower educational attainment disproportionately affected due to limited health and digital literacy [36]. Hospitals could expand grants and funding opportunities to surmount financial hurdles to the smallest rural sites. At the same time, healthcare providers should proactively implement strategies to reach underserved populations and ensure equitable access to essential telehealth services [35].

Reduced travel expenses for patients: Telemedicine can alleviate financial burdens for patients, particularly in rural areas, by mitigating travel-related expenses and enhancing healthcare efficiency and accessibility [26,35]. However, despite its promise, widespread adoption faces challenges such as the initial investment required for telemedicine implementation and the constraints of existing payment models, especially for smaller rural hospitals [35]. Moreover, obstacles, including inadequate reimbursement rates, licensing complexities, infrastructure deficiencies, and regulatory hurdles, can impede telemedicine utilization, particularly among vulnerable groups such as individuals with disabilities [23]. While telemedicine offers opportunities to break down barriers and expand healthcare access, it is imperative to address these challenges to ensure equitable access for all patients, regardless of their geographical location or socioeconomic status [36]. Policymakers and healthcare leaders are actively collaborating to surmount these barriers and ensure that telemedicine remains accessible to all individuals who stand to benefit from its transformative potential [37].

Addressing Sociocultural Barriers

Tailored services for diverse populations: Tailoring telemedicine services to meet the needs of diverse populations is paramount for overcoming sociocultural barriers and ensuring equitable access to healthcare. Healthcare providers must possess cultural competency and an understanding of the social and behavioral nuances inherent in their populations [38]. Redesigning telehealth services to incorporate cultural considerations is crucial, requiring providers to avoid assumptions and actively inquire about patients' comfort levels with technology [38]. When necessary, clear communication, professional interpretation services, and patient accommodation and education are vital components of culturally competent care [39]. Notably, individuals residing in rural areas, those with lower incomes, and those with limited educational attainment may face health and digital literacy challenges, underscoring the need for healthcare providers to proactively reach out and ensure equal access to essential telehealth services [40]. In addition, telemedicine platforms should feature custom functionalities to facilitate healthcare communication for individuals who are deaf or blind, with strict enforcement of web accessibility standards to accommodate persons with disabilities [23].

Language interpretation services: Effective language interpretation services are pivotal in delivering culturally competent care via telemedicine. Leveraging technology such as telemedicine platforms and multilingual chatbots can bridge linguistic divides and promote culturally sensitive interactions [41]. However, it's imperative to acknowledge the digital divide and not assume universal access and proficiency in navigating telehealth services, emphasizing the importance of arranging professional interpretation when needed [39]. Health systems can contribute to addressing language barriers by investing in innovative multilingual digital health technologies and allocating resources for language interpretation services [42]. Equally crucial is implementing cultural competency training for healthcare professionals, fostering an understanding and appreciation of diverse patient needs and perspectives [41]. By integrating these strategies, healthcare providers can ensure that all patients receive high-quality care through telemedicine regardless of their cultural or linguistic backgrounds [43].

Mitigating Infrastructural Limitations

Telemedicine in rural and underserved areas: Telemedicine holds considerable promise for alleviating infrastructural limitations in rural and underserved regions. Nevertheless, numerous challenges hinder its practical implementation in these areas, including inadequate broadband infrastructure, limited access to essential devices, and insufficient telemedicine training for healthcare providers [44,45]. To surmount these obstacles, policymakers must allocate funding for telemedicine infrastructure and provide technical support to facilitate telemedicine deployment in rural communities [44]. In addition, efforts to eliminate financial barriers to telemedicine utilization, such as waiving copayments and offering educational programs, are essential to assist underserved populations in accessing telehealth services [44,46]. Ensuring telemedicine technologies are user-friendly and compatible with the needs of individuals with disabilities and older adults is imperative for promoting inclusivity [44]. Moreover, addressing regulatory and reimbursement challenges is crucial for streamlining the delivery of timely telemedicine care [44,46]. By tackling these barriers, telemedicine can be seamlessly integrated into healthcare delivery systems, mitigating disparities in access and utilization of services in rural and underserved areas [46].

Use of mobile health units: Mobile health units offer a practical solution to infrastructural limitations in telemedicine by deploying telemedicine technology to provide healthcare services in remote or underserved areas [47]. Equipped with telemedicine capabilities and staffed by healthcare providers, these units serve as mobile clinics, delivering essential care directly to needy patients [47]. Moreover, mobile health units play a critical role in emergency response efforts, offering healthcare services during crises or natural disasters when traditional facilities may be inaccessible [48]. However, challenges persist in the implementation of telemedicine in rural areas, including issues related to the acceptability of telemedicine and the digital divide, which may hinder patient access to telehealth services [49]. Healthcare providers and policymakers must collaborate to address these challenges, ensuring that all individuals, regardless of their location, have equitable access to essential telehealth care [48]. Through concerted efforts to overcome barriers and enhance accessibility, mobile health units can effectively extend the reach of telemedicine services to underserved populations, promoting health equity and improving healthcare outcomes in remote areas.

Challenges and limitations

Technological Barriers

Telemedicine encounters various barriers that impede its widespread adoption and efficacy, requiring careful consideration and strategic interventions to overcome. Infrastructure and access barriers present a significant challenge, particularly in rural and underserved areas where limited access to high-speed Internet and digital devices obstructs telemedicine utilization [23]. Operational challenges further compound the issue, as healthcare providers must navigate the complexities of telemedicine devices to maximize their benefits, with the rapid transition to telehealth models during the COVID-19 pandemic exacerbating logistical hurdles [23,50]. Regulatory barriers add another layer of complexity, with varying state rules governing telemedicine practices and potentially restricting physicians' ability to practice across state boundaries based on their licensure. Concerns regarding patient privacy and security risks further complicate regulatory compliance [23,50]. Communication barriers also pose significant challenges, as many telemedicine platforms lack custom features to facilitate healthcare communications for individuals who are deaf or blind. At the same time, a shortage of patient education materials exacerbates difficulties for those with language and literacy challenges [23].

Moreover, the lack of digital literacy is a pressing issue, particularly impacting underserved patients' ability to access and utilize telehealth services effectively [27]. Addressing these multifaceted challenges necessitates proactive measures, with healthcare providers urged to incorporate strategies into their practice to reach underserved populations and ensure equitable access to essential telehealth care [27]. In addition, policy recommendations advocating for removing barriers to telehealth provision and clarity regarding reimbursement for telehealth services are essential to enhance access to care via telemedicine [51]. By addressing these barriers comprehensively, stakeholders can advance telemedicine's potential to expand healthcare access and improve patient outcomes across diverse populations.

Regulatory and Legal Challenges

Navigating the regulatory and legal landscape of telemedicine presents complex challenges, spanning issues such as the corporate practice of medicine, professional entity formation, fee splitting, and adherence to state and federal regulations [52]. Telemedicine platforms must carefully consider myriad legal considerations, encompassing provider licensure, scope of practice, telehealth encounter requirements, and compliance with applicable federal and state laws [52]. Moreover, infrastructure and access barriers, operational complexities, communication obstacles, and legislative hurdles pose additional challenges, potentially impeding the effective delivery of healthcare services via telemedicine [23,53]. Systematically addressing these barriers is imperative to ensure that telemedicine fulfills its potential to enhance access and mitigate healthcare disparities, especially among vulnerable populations such as individuals with disabilities [23]. However, the evolving regulatory landscape and the necessity for adherence to existing laws and regulations pose significant obstacles to the widespread adoption and sustained viability of telemedicine services [54,55]. As such, stakeholders must remain vigilant in navigating these intricate legal and regulatory frameworks to foster telemedicine's continued growth and success as a transformative tool in healthcare delivery.

Patient Acceptance and Adoption

Resistance to change presents a significant barrier to the widespread adoption of telemedicine, particularly among patients residing in rural areas, which may harbor a preference for traditional, in-person care over remote healthcare delivery methods [56,57]. Furthermore, digital literacy and access to technology pose additional hurdles, as patients with lower levels of digital literacy or limited access to technological devices may need help to accept and effectively utilize telemedicine platforms [57,58]. The complexity of systems further compounds these challenges, as patients often favor user-friendly software and may become frustrated with overly intricate telemedicine systems [56]. Moreover, a lack of awareness about telemedicine services exacerbates underutilization, hindering the realization of telemedicine's potential benefits [59]. To address these multifaceted challenges, healthcare providers can explore alternative approaches to delivering telemedicine services, such as offering telephone consultations for patients lacking access to computers or smartphones [59]. In addition, implementing a comprehensive launch strategy incorporating content marketing and social media initiatives can effectively raise awareness about telemedicine services and their availability [59]. By proactively addressing these barriers and implementing strategic interventions, healthcare providers can enhance patient acceptance and utilization of telemedicine, ultimately facilitating improved access to quality healthcare services.

Privacy and Security Concerns

Privacy and security concerns present significant obstacles to the successful implementation of telemedicine. Patients may harbor apprehensions regarding the security of their transmitted information, particularly regarding the confidentiality of their medical data [59,60]. Furthermore, limited access to technology, particularly prevalent in rural or low-income areas, is a deterrent, constraining patients' participation in telemedicine visits [59,60]. Another critical challenge arises from the need for uniform coverage and reimbursement policies across state lines, posing a primary obstacle for healthcare providers seeking telemedicine services across multiple jurisdictions [59]. Moreover, transmitting patient data over the Internet introduces additional security and privacy risks, further complicating the telemedicine landscape [59]. To tackle these challenges effectively, healthcare providers must prioritize the implementation of secure and efficient methods for exchanging electronic health information, ensuring patient privacy and confidentiality [59]. In addition, legislative measures aimed at expanding telemedicine coverage and reimbursement are essential to address disparities in access and incentivize healthcare providers to embrace telemedicine practices [59]. Lastly, proactive efforts to notify patients of available telemedicine services through various communication channels are crucial in promoting awareness and encouraging uptake [60]. By addressing these privacy and security concerns comprehensively, healthcare providers can foster trust and confidence in telemedicine, facilitating its integration into mainstream healthcare delivery.

Future directions and recommendations

Integration of Telemedicine into Healthcare Systems

Advancements in technology are poised to significantly enhance the quality and breadth of telemedicine services by integrating various cutting-edge tools and platforms. These include improved video conferencing platforms, remote monitoring devices, wearable sensors, AI-powered diagnostic tools, and virtual reality applications, all of which promise to augment the capabilities of telemedicine and enrich the patient experience [61]. Moreover, the integration with healthcare technologies is expected to be a key driver in the evolution of telemedicine. It is anticipated to seamlessly integrate with other healthcare technologies, such as electronic health records (EHRs), remote patient monitoring systems, data analytics platforms, and AI algorithms. This integration will facilitate seamless information exchange, real-time monitoring, and data-driven insights, further enhancing the effectiveness and efficiency of telemedicine services [61].

Furthermore, telemedicine is anticipated to expand beyond primary care, extending its reach across various medical specialties and geographic regions. This expansion will enable the delivery of comprehensive and personalized care to patients, regardless of their location or medical needs, thereby broadening the scope and impact of telemedicine in healthcare delivery [61]. Advancements in regulatory and reimbursement policies are expected to play a pivotal role in supporting the integration of telemedicine into mainstream healthcare systems. Further developments in regulations and reimbursement policies are likely to include expanded coverage, standardized guidelines, and increased reimbursement rates for telemedicine services. These advancements will provide essential support and incentives for healthcare providers to adopt and utilize telemedicine effectively [61,62].

Moreover, telemedicine holds promise in addressing healthcare disparities by improving access to underserved populations. By bridging geographical barriers and expanding access to care for underserved communities, including those in rural areas, remote regions, and areas with limited healthcare resources, telemedicine can significantly reduce healthcare disparities and improve health outcomes for vulnerable populations [61,62]. While the future of telemedicine holds tremendous promise for revolutionizing healthcare delivery and enhancing patient outcomes, it is imperative to address various challenges, such as ensuring equitable access, safeguarding patient privacy and data security, and navigating regulatory considerations across jurisdictions. By addressing these challenges proactively, stakeholders can harness the full potential of telemedicine to transform healthcare delivery and improve access to quality care for all.

Policy Initiatives to Promote Telemedicine Accessibility

Advancements in regulatory and reimbursement policies constitute a crucial aspect of fostering telemedicine services' widespread adoption and sustainability. Further developments in regulations and reimbursement policies are essential to support and incentivize telemedicine practices. This includes expanded coverage, establishing standardized guidelines, and increased reimbursement rates for telemedicine services [61]. Improving access to underserved populations is paramount in addressing disparities in healthcare access. This involves tackling various barriers, including infrastructure limitations, access challenges, operational complexities, regulatory constraints, and communication obstacles. By addressing these barriers comprehensively, healthcare providers can enhance access to care for underserved populations, particularly those in rural areas and remote communities [23,61].

Integration with other healthcare technologies is essential for optimizing the effectiveness and efficiency of telemedicine. By integrating telemedicine with other healthcare technologies, such as EHRs, remote patient monitoring systems, data analytics platforms, and AI algorithms, healthcare providers can facilitate more comprehensive and coordinated care delivery [61]. Addressing communication and legislative barriers for persons with disabilities is imperative for promoting inclusivity in telemedicine services. This involves enforcing web accessibility standards on telemedicine platforms, addressing communication barriers for individuals who are deaf or blind, and ensuring that legislative frameworks such as the Americans with Disabilities Act are adapted to accommodate virtual spaces and services like telemedicine [23]. Minimizing barriers and challenges is essential to ensure the successful implementation of telemedicine care. This includes mitigating risks such as privacy loss, confidentiality breaches, fraud, and abuse. While telemedicine can potentially improve patient care access, it is crucial to proactively address these challenges and barriers to ensure equitable access and quality care for all individuals, including those in underserved populations and persons with disabilities [36,63].

Research Priorities for Further Understanding the Impact

Research in telemedicine should prioritize several key areas to enhance healthcare accessibility and ensure equitable healthcare delivery. First, efforts should be directed toward promoting health equity by addressing the digital divide and improving access to telehealth for underserved populations, including individuals with lower incomes, limited health literacy, and rural residents [27]. Understanding user experience and utilization factors is crucial for optimizing telemedicine adoption rates and patient satisfaction, necessitating research into the determinants of patient utilization and strategies to enhance the user experience [64]. In addition, there is a pressing need for further investigation into the impact of telemedicine on chronic care management and mental health services, providing insights to guide future developments in these critical healthcare domains [64].

Policy and regulation represent another significant focus area for research, with studies needed to evaluate the impact of policy changes on telemedicine adoption and to identify regulatory barriers that may impede its effectiveness [63]. Moreover, exploring provider perspectives is essential for understanding healthcare providers' experiences and attitudes toward incorporating telehealth into their practice, particularly in reaching underserved patient populations [27]. By addressing these critical areas through rigorous research endeavors, future studies can contribute to a more comprehensive understanding of the impact of telemedicine on healthcare accessibility and inform the development of effective strategies to maximize its benefits for all patients.

Training and Education for Healthcare Professionals

The future landscape of healthcare education, particularly for healthcare professionals, is undergoing a profound transformation driven by the convergence of telemedicine and eLearning technologies. Telemedicine stands at the forefront of this transformation, potentially revolutionizing healthcare delivery by enhancing access and alleviating patient and provider burdens [65]. Concurrently, eLearning platforms are increasingly becoming invaluable tools in graduate and postgraduate healthcare professional education, providing institutions with innovative means to impart knowledge on various topics flexibly and conveniently, irrespective of geographical constraints [66]. Telemedicine and eLearning synergistically present opportunities to advance rural workforce education and training, bridge geographical gaps between specialists, and bolster the capabilities of healthcare providers in remote areas to manage complex cases effectively [66]. Nonetheless, several challenges must be addressed to realize the full potential of these educational modalities. Ensuring the competency of faculty and instructors in telehealth practices and maintaining educational quality amidst rapid digital advancements emerge as prominent concerns [67]. To tackle these challenges, institutions can explore collaborative partnerships with telehealth providers, seek grant funding opportunities, and develop specialized assessment tools tailored to assess telehealth competencies effectively [67]. By integrating telehealth methodologies into educational systems and adopting proactive measures to address associated challenges, healthcare professionals can be adequately prepared for the evolving landscape of healthcare delivery [67]. This concerted effort toward leveraging telemedicine and eLearning in healthcare education underscores their pivotal role in shaping the future of healthcare professionals' training and readiness for the dynamic healthcare environment ahead.

## Conclusions

The review underscores telemedicine's pivotal role in enhancing healthcare accessibility. Telemedicine effectively addresses various barriers hindering individuals ' access to healthcare services through remote consultations, monitoring, and diagnosis facilitated by technological advancements. Geographical accessibility is vastly improved as telemedicine extends healthcare reach to remote and underserved areas, while temporal accessibility is enhanced through round-the-clock availability. Moreover, telemedicine effectively mitigates financial, sociocultural, and infrastructural barriers, promoting health equity and improving health outcomes. Its ability to streamline healthcare delivery systems reduces costs and fosters efficiency. Moving forward, stakeholders in the healthcare and technology sectors must collaborate to realize the full potential of telemedicine. Policymakers should enact regulations that support telemedicine adoption while ensuring patient safety and privacy. Healthcare providers must integrate telemedicine into their practices, embracing it as a complement to traditional care delivery models. Concurrently, technology companies should continue innovating to develop intuitive telemedicine platforms that cater to the needs of both providers and patients. Through concerted efforts, stakeholders can harness telemedicine's transformative potential, advancing healthcare accessibility and enhancing the health and well-being of individuals globally.
